# Effects of green tea supplementation on inflammation markers, antioxidant status and blood pressure in NaCl-induced hypertensive rat model

**DOI:** 10.1080/16546628.2017.1295525

**Published:** 2017-03-03

**Authors:** Monika Szulińska, Marta Stępień, Matylda Kręgielska-Narożna, Joanna Suliburska, Damian Skrypnik, Monika Bąk-Sosnowska, Magdalena Kujawska-Łuczak, Małgorzata Grzymisławska, Paweł Bogdański

**Affiliations:** ^a^ Department of Education and Obesity Treatment and Metabolic Disorders, Poznań University of Medical Sciences, Poznań, Poland; ^b^ Department of Human Nutrition and Hygiene, Poznań University of Life Sciences, Poznań, Poland; ^c^ Department of Internal Medicine, Metabolic Disorders and Hypertension, Poznań University of Medical Sciences, Poznań, Poland; ^d^ Department of Psychology, Chair of Philosophy and Humanities, School of Health Sciences in Katowice, Medical University of Silesia, Katowice, Poland; ^e^ Department of Anatomy, Poznań University of Medical Sciences, Poznań, Poland

**Keywords:** Diet supplementation, hypertension, inflammatory status, antioxidants, oxidative stress

## Abstract

**Background:** Recent studies indicate the important role of chronic inflammation and oxidative stress in the pathogenesis of hypertension. Green tea, due to the high content of catechins, shows high antioxidant activity.

**Objective:** To determine the effect of supplementation with green tea extract on the blood pressure, on the concentration of selected parameters of inflammation and antioxidant status in the model of high-sodium-diet induced hypertension.

**Design:** The study lasted 42 days. The experimental population consisted of 30 rats. The rats were divided into three groups. The rats in the control group were fed a standard diet with 35 g of NaCl per kg of diet, in the second group hypertensive rats were fed a standard diet with NaCl (35 g/kg diet) and with an extract of green tea (2 g/kg diet). The third group consisted of hypertensive rats fed a standard diet with NaCl (35 g/kg diet), and 4 g of green tea extract/kg diet.

**Results:** Supplementation with green tea had no effect on body mass of rats on a high-sodium diet. At the end of the experiment systolic blood pressures in SH2 and SH4 groups were significantly lower than in the control group SK. The SH4 group was characterized by a significantly lower diastolic blood pressure value and concentration of TNF-α in comparison to the SK group. The rats from both SH2 and SH4 groups were characterized by higher total antioxidant status values compared to the control group.

**Discussion:** The mechanism of the beneficial effects of green tea on blood pressure is not clear, but it is believed that it is related to its omnidirectional properties.

**Conclusions:** Supplementation of green tea has a beneficial effect on blood pressure, markers of inflammation and antioxidant status in an experimental model of hypertension.

## Introduction and background

While it was initially considered that the increased activity of the intravascular inflammatory process is the result of elevated arterial pressure, recent studies also indicate the important role of chronic inflammation and oxidative stress in the pathogenesis of hypertension.[1] It is postulated that one of the important mechanisms leading to activation of the immune system in hypertension is the immune response to neoantigens produced as a result of oxidative stress. Neoantigens lead to T-cell activation, release of T-cell adhesion molecules including intercellular adhesion molecule-1 (ICAM-1) and vascular cell-adhesion molecule-1 (VCAM-1) and their receptors, as well as increased production of proinflammatory cytokines: interleukin-1 (IL-1), interleukin-17 (IL-17), interleukin-6 (IL-6) and tumor necrosis factor-α (TNF-α).[2] The most common parameter suggesting the presence of increased inflammatory process is C-reactive protein (CRP). A correlation has been shown between relatively elevated CRP levels and blood pressure.[3,4]

Oxidative stress is another important element in the pathogenesis of hypertension. The organism has the ability to remove reactive oxygen species (ROS) and defense against oxidative stress through antioxidant enzymes. These include, among others, superoxide dismutase (SOD), glutathione peroxidase, catalase and peroxides. It is believed that oxidative stress plays an important role in the development of hypertension.[5] The mechanism of hypertension induction by free radicals is complex and includes: reduction of vascular-expanding bioavailability of nitric oxide (NO), vasoconstrictor effect of ONOO- anion, impaired vasodilatation as a result of membrane lipid peroxidation, stimulation of endothelin production and vascular smooth muscle proliferation, and the impact on volemia by increasing the resorption of sodium in renal tubules.[5] ROS also directly affect the inflammatory response responsible for the further increase in blood pressure.[6] The key point in prevention and treatment of patients with hypertension is an effective blood pressure lowering and rectification of all modifiable risk factors to reduce overall cardiovascular risk. Antioxidants as a part of the diet are an integral part of nonpharmacological treatment of hypertension.

The best-known antioxidants, besides vitamins C and E, are β-carotene, ubichinion, glutathione, flavonoids, taurine and phytoestrogens. Several experimental studies as well as clinical studies suggest a beneficial effect of antioxidant supplementation in patients with cardiovascular disease. Their mechanism of action is based primarily on reducing oxidative stress, enhancing NO synthesis, reducing LDL oxidation, inhibiting the growth of smooth muscle cells of blood vessels and reducing blood pressure.[7] It has been observed that the administration of antioxidants: 200 mg of zinc, 500 mg of vitamin C, 600 IU of vitamin E and 30 mg of β-carotene for eight weeks decreased the blood pressure both in hypertensive patients and patients with normal blood pressure.[8] In the group of polyphenols and antioxidant vitamins the most effective are catechins – the compounds found in green tea.

The antioxidant activity of tea depends largely on the quantity and quality of polyphenolic compounds in it. Green tea, due to its high catechin content, representing approximately 78% of the polyphenol fraction, showed the highest antioxidant activity among all kinds of teas in an assay with 1,1-diphenyl-2-picrylhydrazyl reagent (DPPH).[9] Green tea catechins effectively eliminate free radicals, which are involved in the pathogenesis of many diseases. The results indicate that catechins contained in green tea have the ability to prevent the development of atherosclerosis and hypertension, and to reduce the mortality due to cardiovascular diseases.[10] The participation of oxygen free radicals and inflammation in the pathogenesis of hypertension justifies the use of a diet rich in antioxidants in these patients. Despite numerous experimental studies, convincing evidence of the antihypertensive and anti-inflammatory impact of green tea is still needed. We have carried out studies in normotensive Wistar rats subjected to increased sodium intake in the diet, as a model reflecting hypertension with the features of human metabolic syndrome, and the results allow for new insights into the mechanisms of the action of green tea and its potential beneficial effects both in the prevention of arterial hypertension and treatment of patients with this condition.

The aim of this study was to determine the effect of supplementation of the diet with green tea extract on blood pressure, the concentration of selected parameters of inflammation, and antioxidant status, in an experimental model of high-sodium-diet induced hypertension.

## Material and methods

### Animals

All animal procedures were performed according to approved protocols and to Poznan University of Life Sciences guidelines. The experiment conformed to the legal requirements in Poland as well as with the European Communities Council Directive of 24 November 1986 and the National Institute of Health Guide (National Institute of Health Publications No. 80–23, Revised 1978) for the care and use of Laboratory Animals for experimental procedure. This study was approved by the local Ethics Committee (approval no. 20/2011, year 2011 and 28/2012, year 2012). The experiment was performed on male Wistar rats (eight weeks old), derived from the Department of Toxicology, University of Medical Sciences, Poznań. The rats adapted to laboratory conditions during the first five days. The mean body mass of the rats was 193 ± 2 g. The animals were housed individually in stainless steel cages coated with metal-free enamel, and kept under controlled room conditions: temperature (21°C), humidity (55–65%), 12/12 light/dark cycle.

### Experimental design

Thirty animals were randomly assigned to three groups of 10 rats each: the control group (SK), a group with low amount of green tea extract (SH2), and a group with high amount of green tea extract (SH4). All rats were fed a standard diet (maintenance diet for rats 1320, Altromin, Lage, Germany), whose full composition is presented in Table 1, with added sodium chloride 35 g per kg of diet. In the diet of SH2 and SH4 groups, extract of green tea was added at a rate of 2 and 4 g/kg diet, respectively. The intake of the diet was monitored daily. The rats were weighed once a week. The animals are allowed to eat diet and drink distilled water for 42 days ad libitum.

**Table 1.** Body mass of rats at the end of the experiment.

	Group	*N*	Mean	Median	Minimum	Maximum	SD	*p*-value
**Body mass(g)**	SK	10	287.50	286.00	265.00	315.00	16.22	0.7174
	SH2	10	282.90	282.00	267.00	309.00	14.70	
	SH4	10	286.70	288.00	259.00	312.00	16.98	

SD- standard deviation

### Serum collection

At the end of the experimental period, the animals were weighed and anesthetized with a sodium thiopental injection (40 mg/kg body mass). Blood was collected from the rats following 12 h of fasting. The blood samples were collected by cardiac puncture in serum-separated tubes to obtain serum. The coagulated blood was left to clot at room temperature for 30 min, and then centrifuged for 15 min at 2000 rpm at 4°C; the supernatant fluid was then separated and stored frozen (–80ºC) for analysis.

### Biochemical measurements

The concentration of TNF-alpha in the blood serum was measured by enzyme immunoassay (enzyme-linked immunosorbent assay) (R&D Systems, Inc., Minneapolis, MN, USA). Total antioxidant status (TAS) was measured using a TAS Randox kit (Randox Laboratories, Ltd, Crumlin, UK) and spectrophotometry (SPECORD M40; Carl Zeiss Jena, Germany). Serum CRP level was determined by ELISA (R&D system, Minneapolis, Minnesota, USA).

### Statistical analysis

Detailed statistical analysis was performed using Statistica for Windows 10.0 (StatSoft, Kraków, Poland). The normality of the variables’ distribution was verified using the Shapiro–Wilk test of normality. The results were expressed as arithmetic means with standard deviation. One-way analysis of variance (ANOVA) was used to compare the data between groups. The correlations between biochemical variables were calculated using the Spearman test. The significance was set at the *p* < 0.05 level.

## Results

It was found that supplementation of diet with green tea extract had no effect on body mass of rats in each groups. The body mass of rats after the end of the experiment did not differ significantly between the groups (Table 1).

### Blood pressure

The mean systolic blood pressure (SBP) and diastolic blood pressure (DBP) in rats at the beginning of the experiment was 113.9 ± 4.5 mmHg and 72.2 ± 3.4 mmHg respectively. At the end of the experiment SBP in SH2 and SH4 groups were significantly lower than in the control group SK (*p* < 0.001 and *p* < 0.05 respectively). In addition, the SH4 group was characterized by a significantly lower DBP value in comparison to the SK group (*p* < 0.01). DBP values did not differ significantly between the groups SH2 and SK. Detailed data are shown in Table 2.

**Table 2.** Blood pressure values in each group of rats at the end of the experiment.

	Group	*N*	Mean	Median	Minimum	Maximum	SD	*p*-value
**SBP(mmHg)**	SK	10	154.5	155.8	117.7	172.3	16.7	0.0066
	SH2	10	126.0	131.9	86.1	160.8	22.6	
	SH4	10	134.5	135.0	117.7	152.3	10.1	
**DBP(mmHg)**	SK	10	95.2	95.0	90.0	102.3	4.4	0.0033
	SH2	10	82.5	76.9	53.8	116.9	20.5	
	SH4	10	70.6	69.6	46.1	90.8	12.3	

The parameters in all groups were consistent with normal distribution (Shapiro–Wilk test). SBP, systolic blood pressure; DBP, diastolic blood pressure; SD, standard deviation.

### The concentration of inflammatory parameters and antioxidant status

Concentrations of TNF-α were significantly lower in the SH4 group compared to SK group (*p* < 0.01). There was no statistically significant difference in concentration of this parameter between the SH2 and SK groups (Table 3). CRP levels did not differ between the groups (Table 3). The rats from both groups receiving the green tea extract SH2 and SH4 were characterized by significantly higher TAS values compared to the control group SK (*p* < 0.001 and *p* < 0.01 respectively). Furthermore, a significantly higher TAS values in the SH4 group compared to the SH2 group was revealed (*p* < 0.05). The data are presented in Table 3. Analysis of correlation was performed on the entire population of rats, and it concerned the mutual relationship between the investigated parameters. A statistical significant relationship is presented below. In the rat population covering all three groups (SK, SH2, SH4) together, statistically significant negative correlations between the levels of TAS and SBP, DBP, and TNF-α were found. Detailed data are presented in Table 4.

**Table 3.** The concentrations of inflammatory and oxidative stress parameters in a model of hypertension in each group of rats at the end of the experiment.

	Group	*N*	Mean	Median	Mininum	Maximum	SD	*p*-value
**TNF-α (ng ml^–1^)**	SK	10	3.08	3.37	2.02	4.10	0.76	0.0095
	SH2	10	2.63	2.70	2.00	3.20	0.36	
	SH4	10	2.20	1.98	1.76	2.91	0.43	
**CRP (ng ml^–1^)**	SK	10	82.20	83.50	75.00	87.00	3.77	0.07
	SH2	10	79.80	79.50	76.00	84.00	2.74	
	SH4	10	78.70	78.50	75.00	84.00	3.16	
**TAS (mM)**	SK	10	1.09	0.95	0.88	1.60	0.27	0.0001
	SH2	10	1.69	1.80	1.22	1.93	0.25	
	SH4	10	2.23	2.23	2.08	2.31	0.07	

CRP levels in all groups were consistent with normal distribution (Shapiro–Wilk test). Part of TNF-α and TAS levels were not consistent with a normal distribution (Shapiro–Wilk test). TNF-α, tumor necrosis factor; CRP, C-reactive protein; TAS, plasma total antioxidant status; SD, standard deviation.

**Table 4.** Significant correlations between TAS and SBP, DBP and TNF-α in the entire population of rats.

Correlated parameters	*N*	The correlation coefficient R	*p*
**TAS (mM) & SBP (mmHg)**	30	−0.4005	0.0283
**TAS (mM) & DBP (mmHg)**	30	−0.6460	0.0001
**TAS (mM) & TNF-α (ng ml^–1^)**	30	−0.5083	0.0041

TAS estimated in relation to the total of all three groups were not consistent with a normal distribution (Shapiro–Wilk test). TAS, plasma total antioxidant status; SBP, systolic blood pressure; DBP, diastolic blood pressure; TNF-α, tumor necrosis factor.

## Discussion

In this study, statistically significant lower values of SBP and DBP in the group of animals treated with green tea extract as compared to the control group were revealed. The results of most experimental studies confirm the positive effect of supplementation of green tea on blood pressure in various animal models. Potenza et al. [11] found that a supply of green tea causes a significant reduction in blood pressure in spontaneously hypertensive rat (SHR), which was comparable with the effect of antihypertensive pharmacological therapy with the use of enalapril. Similar results were found by Negishi et al. [12] in rats with spontaneous hypertension prone to stroke (spontaneously hypertensive stroke prone rats; SHRSP) and by Ihm et al. [13] who gave a green tea extract to rats with metabolic syndrome.

The mechanism of the beneficial effects of green tea on blood pressure is not clear, but it is believed that it is related to its omnidirectional properties. Green tea extract regulates vascular homeostasis by its influence on the production of vasoconstrictive substances including angiotensin II, prostaglandins, endothelin-1 as well as vasodilating substances such as prostacyclin.[14]

The results of numerous studies have shown a clear connection between hypertension and relatively elevated CRP levels. There is also convincing evidence for an association of TNF-α, a key pro-inflammatory cytokine, with hypertension.[15] The increased activity of inflammatory processes observed in hypertension results partly from mechanical damage of the vascular endothelium due to chronically elevated levels of blood pressure, as well as metabolic disorders frequently co-morbid with hypertension. The influence of these factors in the severity of inflammation is confirmed in this paper by positive correlations between markers of inflammation and SBP.

In the presented study, there was a statistically significant reduction in the levels of TNF-α in the group of rats treated with 4 g of green tea extract as compared to the control group. Other experimental and clinical observations provide evidence for the anti-inflammatory effects of green tea. In the study of Cao et al. [16], the green tea extract significantly influenced the reduction of TNF-α mRNA level in Wistar rats fed with a high fructose diet. Chung et al. [17] have shown anti-inflammatory properties of green tea in Wistar rats fed with a high fat diet supplemented with 0, 1 and 2% green tea extract for a period of eight weeks.

In our study there was no difference in the level of CRP between groups of rats consuming green tea extract and the control group. Li et al. [18] observed that epigallocatechin gallate (EGCG) inhibits the production of CRP induced by angiotensin II and IL-6 in macrophages by decreasing the production of free oxygen radicals. The same authors showed that consumption of four cups of green tea per day for four weeks significantly reduced the levels of CRP in smokers, suggesting anti-inflammatory action of catechins in humans.[18] Yilmaz et al. [19], in a clinical trial on a group of 244 patients with primary hypertension, showed that a diet with a high content of salt exacerbates inflammation expressed by increased CRP concentration independently from blood pressure.

It was observed that green tea catechins may modulate the pro-inflammatory signaling pathways by suppressing the activation of transcription factors. The study of Riegsecker et al. [20] demonstrated that the supply of EGCG can minimize damage of endothelial cells, decreasing production of IL-6 and TNF-α by inhibiting the action of transcription factors activator protein 1 (AP-1) and nuclear factor kappa-light-chain-enhancer of activated B cells (NF-kB). A similar mechanism of action of catechins is observed by Wang et al., [22] in the primary cells of the vascular endothelium isolated from human umbilical vein endothelial cells (HUVEC) and by Cavet et al. [21] in human corneal epithelial cells (HCEpiC). Effects of EGCG on NF-kB and other types of cells are based on multiple mechanisms including the inhibition of IkB kinase, IkB phosphorylation, acetylation p65NFκB and decreasing the binding activity of NF-kB to DNA. Inhibition of factor AP-1 by EGCG it is mainly due to the effects on mitogen-activated protein kinase (MAPK) signaling pathways and activity of AP-1 to DNA binding.[22]

Chronic high blood pressure is one of the factors that significantly increase the severity of oxidative stress.[23] In this study, the importance of modeling oxidative stress by elevated blood pressure values is confirmed by the negative correlation between the level of TAS, which reflects plasma TAS, and SBP and DBP. It should be noted that oxidative stress is closely linked to inflammation.[24,25] In the presented work it is reflected by a negative correlation between TAS and TNF-α.

Green tea catechins, as one of the strongest antioxidants, protect cells from the damaging effects of ROS. Antioxidant activity of green tea was confirmed in our study. A similar beneficial antioxidant effect of green tea was shown by Ihm et al. [13] in a model of metabolic syndrome in rats. Consumption of green tea significantly reversed the adverse impact of ethanol intoxication in rats expressed by TAS reduction in erythrocytes. The effect of long-term consumption of green tea was protection of erythrocyte membranes against the effects of aging and/or toxic effects of ethanol.[26] In addition, Kager et al. [27] showed that green tea in a dose equivalent to 500 ml in human protects the DNA of lymphocytes and internal organs from oxidative damage in rats, while a smaller dose, equivalent to 100 ml of green tea in human per day did not present this property.

The consumption of green tea also induces a positive change in the level of antioxidative status in humans. Drinking green tea in an amount of four brewing sachets containing 1.5 g of green tea per day for 12 weeks increased antioxidative capacity of saliva by 42% in a population of the elderly.[28,29]

The mechanism of antioxidant action of catechins is based on: direct inactivation of reactive oxygen and nitrogen, the chelation of transition metals, the regeneration of antioxidants i.e. β-carotene or α-tocopherol, and inhibition of pro-oxidant enzymes.[23] Experimental and clinical data indicate that green tea catechins, due to their antioxidant properties, increase the antioxidant capacity of plasma,[6,30] reduce the concentration of lipid peroxidation products markers [31] and oxidative stress in erythrocytes, and the level of oxidative DNA damage markers in smokers.[32,33] Green tea reduces oxidative stress and production of ROS by inhibiting oxidative enzymes and activation of antioxidant enzymes.[34] Studies have shown that green tea reduces endothelial reduced nicotinamide adenine dinucleotide phosphate (NADPH) oxidase activity, reduces the production of superoxide anion O_2_^−^ and protects NO molecules against oxidation, increasing NO bioavailability.[34] The authors also confirm that green tea polyphenols reduce production of ROS, by significant increase of catalase expression – an enzyme catalyzing the degradation of hydrogen peroxide.[35]

Further studies to confirm the effect of green tea supplementation on cardiometabolic parameters are needed. Confirmation of the positive effect of many dietary supplements requires both experimental and clinical studies to be conducted.

### Study limitations

The main drawback of the study results from the lack of non-high-sodium diet feed control group. The influence of high-sodium diet on the investigated parameters remains unclear.

## Conclusion

In conclusion, supplementation of green tea has a beneficial effect on blood pressure and antioxidative status in an experimental model of high-sodium-diet induced hypertension.
